# Characterization and Development of EST-SSRs by Deep Transcriptome Sequencing in Chinese Cabbage (*Brassica rapa* L. ssp. *pekinensis*)

**DOI:** 10.1155/2015/473028

**Published:** 2015-10-04

**Authors:** Qian Ding, Jingjuan Li, Fengde Wang, Yihui Zhang, Huayin Li, Jiannong Zhang, Jianwei Gao

**Affiliations:** ^1^College of Horticulture, Gansu Agricultural University, Lanzhou 730070, China; ^2^Institute of Vegetables and Flowers, Shandong Academy of Agricultural Sciences and Shandong Key Laboratory of Greenhouse Vegetable Biology and Shandong Branch of National Vegetable Improvement Center, Jinan 250100, China

## Abstract

Simple sequence repeats (SSRs) are among the most important markers for population analysis and have been widely used in plant genetic mapping and molecular breeding. Expressed sequence tag-SSR (EST-SSR) markers, located in the coding regions, are potentially more efficient for QTL mapping, gene targeting, and marker-assisted breeding. In this study, we investigated 51,694 nonredundant unigenes, assembled from clean reads from deep transcriptome sequencing with a Solexa/Illumina platform, for identification and development of EST-SSRs in Chinese cabbage. In total, 10,420 EST-SSRs with over 12 bp were identified and characterized, among which 2744 EST-SSRs are new and 2317 are known ones showing polymorphism with previously reported SSRs. A total of 7877 PCR primer pairs for 1561 EST-SSR loci were designed, and primer pairs for twenty-four EST-SSRs were selected for primer evaluation. In nineteen EST-SSR loci (79.2%), amplicons were successfully generated with high quality. Seventeen (89.5%) showed polymorphism in twenty-four cultivars of Chinese cabbage. The polymorphic alleles of each polymorphic locus were sequenced, and the results showed that most polymorphisms were due to variations of SSR repeat motifs. The EST-SSRs identified and characterized in this study have important implications for developing new tools for genetics and molecular breeding in Chinese cabbage.

## 1. Introduction

Chinese cabbage (*Brassica rapa* L. ssp.* pekinensis*) is a diploid (2*n* = 2*x* = 20) dicot with a genomic size of 550 Mb (http://www.brassica.info/resource/). It is a subspecies of* B. rapa* with the A genome [1]. The species originated in China and now has become one of the most important and widely cultivated leaf vegetables in Asia. Chinese cabbage has rosette leaves (RLs) and folding leaves (FLs). The tight leafy head is the main edible part. After a long history of domestication, Chinese cabbage evolves into different cultivars with a variety of characteristics, such as rosette leaf morphology, heading leaf morphology, leafy head shape, size, and structure, flowering time, nutrient composition, and resistance to biotic and abiotic. A better understanding of the molecular mechanism of evolution of Chinese cabbage and further development of marker-assisted selection (MAS) will accelerate the selection process of improved cultivars to meet the growing consumers and environmental needs. Although progress has been made in underlining the molecular mechanism [2–5], many aspects are still unclear.

Molecular markers have been widely used to study the genetic basis of important traits and map regulatory genes in plants. Markers tightly linked with important agronomic traits can potentially be used for molecular breeding to develop improved cultivars. Many molecular markers and genetic maps of Chinese cabbage have been reported previously [6–25]. However, there is still a great need to develop novel molecular markers for construction of high-density linkage maps for genetics and molecular studies of important traits in Chinese cabbage.

Simple sequence repeat (SSR) markers or microsatellite markers are among the most important markers in plants. SSRs have been widely used in genetic mapping and molecular breeding in plants because they are highly abundant and have significant polymorphism. Other factors, like accessibility for detection, reliability, and codominance, also make them perfect markers for such purposes [26]. SSRs found in transcribed sequences are called expressed sequence-simple sequence repeats (EST-SSRs). Compared with genomic-SSRs detected in noncoding sequences, EST-SSRs are more efficient for QTL mapping, gene targeting, and MAS [27]. As transcribed sequences are more conserved than noncoding sequences, the transferability of EST-SSRs is better than genomic-SSRs [28–30], which can be utilized for cross genome comparison and evolutionary analysis [27, 31]. Additionally, abundant ESTs were generated in recent years with the development of next-generation sequencing approaches, making identification of EST-SSRs more practical and cost-efficient [32]. Many EST-SSRs have been identified in Chinese cabbage [16, 20, 25, 33–36]. Because whole genome sequencing of Chinese cabbage is still underway, new EST-SSRs could also be identified for further studies such as high-density genetic linkage map construction, gene/QTL mapping, and cultivar identification.

In our previous study, the whole transcriptomes were analyzed for the rosette leaves and folding leaves of a typical heading Chinese cabbage, namely, FuShanBaoTou, using a Solexa/Illumina RNA-Seq platform, and a large-scale EST database was generated [37]. In this study, we further assembled those ESTs from the RL and FL libraries into nonredundant unigenes. A total of 10,420 EST-SSRs were identified, among which 2744 EST-SSRs are detected for the first time, according to the SSR marker database for* Brassica* (http://oilcrops.info/SSRdb). We characterized these identified EST-SSRs and designed 7877 PCR primer pairs for 1561 EST-SSRs. Furthermore, serving as a validation purpose, we tested polymorphisms of 24 EST-SSRs. We expect this study can pave the road for further investigation of new EST-SSR markers and for construction of high-density genetic maps.

## 2. Materials and Methods

### 2.1. Plant Materials

For EST-SSR identification and primer design, a typical heading Chinese cabbage, namely, FuShanBaoTou, was used in this study. For primer assessment and SSR polymorphism analysis, a panel of twenty-four cultivars of Chinese cabbage was used, including nineteen morphologically diverse cultivars of* Brassica rapa *L. ssp*. pekinensis* (*B. pekinensis* L.) and five* Brassica rapa *L.* chinensis (B. chinensis *L.). All plants were grown in a greenhouse with 16/8 photoperiod at 22 ± 2°C. Leaves were collected after they were grown for two weeks from ten seedlings of each cultivar and were pooled together for DNA extraction.

### 2.2. De Novo Assembly

We assembled the clean read dataset presented by Wang et al. [37] from the RL and FL libraries according to the methods described by Wang et al. [38] using the Trinity software (http://trinityrnaseq.sourceforge.net/). Contigs and unigenes were obtained from these two libraries, respectively. Redundant sequences were removed and overlapping unigenes were assembled into continuous sequences by the TIGR Gene Indices Clustering (TGICL) tools [39]. Similarity was set at 94% and an overlap length was set at 100 bp.

### 2.3. Identification of EST-Derived SSRs and Primer Design

SSRs were detected with the MicroSAtellite software (MISA; http://pgrc.ipk-gatersleben.de/misa/). Parameters were set with a minimum number of 12, 6, 5, 5, 4, and 4 repeat units for identification of mono-, di-, tri-, tetra-, penta-, and hexanucleotide motifs, respectively. Primers were designed using primer 3 with no SSR allowed in primers. Primer length ranged from 18 to 28 bp (with an optimality at 23). Annealing temperature was set at 55–65°C (with an optimality at 60°C). The size of a PCR product ranged from 80 to 300 bp.

### 2.4. Mapping EST-SSRs

The physical positions of the EST-SSRs identified in the study were determined by aligning the SSRs and flanking sequences (50 bp at each side) to the* Brassica rapa* (Chiifu-401) reference genome (http://brassicadb.org/brad/) using BLASTN. New EST-SSRs were identified by comparing with previously reported SSRs in the SSR marker database for* Brassica* (http://oilcrops.info/SSRdb) [25].

### 2.5. SSR Amplification and SSR Polymorphism Analysis

DNA was extracted following a CTAB DNA extraction protocol [40]. The DNA sample of the Chinese cabbage FuShanBaoTou was used as template to detect the availability of SSR primers designed above. The DNA samples of those aforementioned twenty-four cultivars of Chinese cabbage were used as templates for SSR polymorphism analysis. The polymorphisms of EST-SSRs were validated by 6% denaturing polyacrylamide gel electrophoresis, 12% nondenaturing polyacrylamide gel electrophoresis, and sequencing.

## 3. Results

### 3.1. De Novo Assembly

High quality clean read data from the RL and FL libraries by Wang et al. [37] were assembled using the Trinity software package [41]. A total of 99,684 and 95,411 contigs were obtained, with an average length of 333 and 342 bp and a median length (N50) of 531 and 536 bp, from the RL and FL libraries, respectively (Table 1).

**Figure 1:**
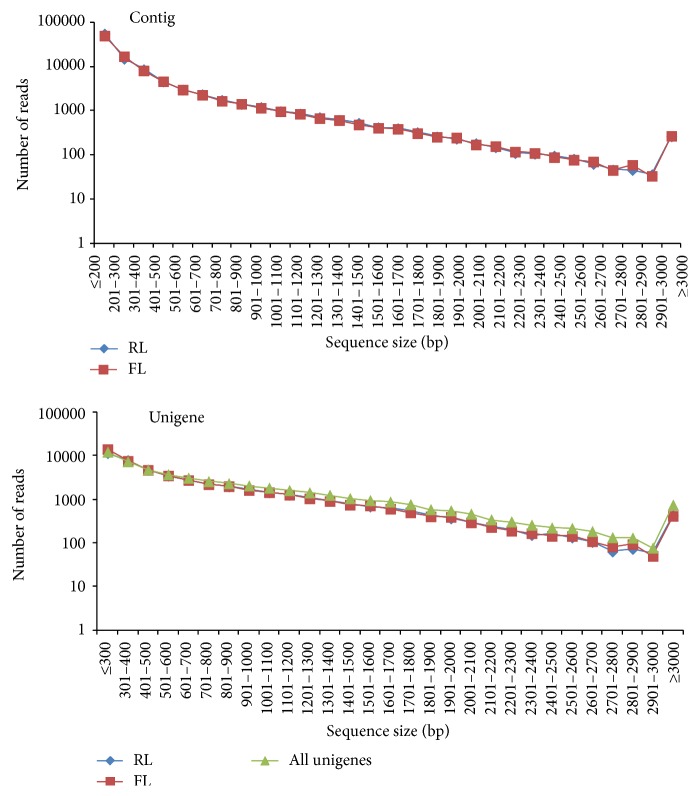
Size distribution of the assembled contigs and unigenes in RL and FL libraries.

Contigs from the same transcript were detected with paired-end reads, as well as the distances between these contigs. Using the Trinity software package, we assembled these contigs into unigenes, in which Ns were removed. These unigenes were set to be not extendable on either end of the sequences. A total of 46,294 and 48,473 unigenes from the RL and FL libraries were obtained with an average length of 707 and 680 bp and a median length (N50) of 1000 and 980 bp, respectively (Table 1). Size distribution of the contigs and unigenes is consistent with the RL and FL libraries as shown in Figure 1, indicating that our Illumina sequencing solution is reliable and reproducible. Unigenes from the two samples were combined; redundant unigenes were removed; and the rest was assembled with TGICL [39] to form a single dataset, which represents 40.7 Mb of sequence and contains a total of 51,694 nonredundant unigenes, with an average read length of 788 bp, and a median read length (N50) of 1154 bp (Table 1). The sequences of the unigenes are listed in Table s1 (see Supplementary Material available online at http://dx.doi.org/10.1155/2015/473028).

The length of 24,271 nonredundant unigenes (46.95%) is between 200 and 500 bp; the length of 13,613 (26.33%) is between 501 and 1,000 bp, and the length of 13,810 (26.72%) is longer than 1,000 bp (Figure 1).

### 3.2. Characterization of EST-SSRs in Chinese Cabbage

A total of 10420 EST-SSRs were detected with the MicroSAtellite software (MISA; http://pgrc.ipk-gatersleben.de/misa/) in 8571 unigenes, accounting for 16.6% of total nonredundant unigenes (Tables 2 and s2). The mean SSR density is one per 3.9 Kb, corresponding to one for every 5.0 nonredundant unigenes. 1502 unigenes (17.5%) harbored more than one SSR and 666 SSRs (6.4%) were present in compound formation that had more than one repeat type (Table 2).

**Figure 2:**
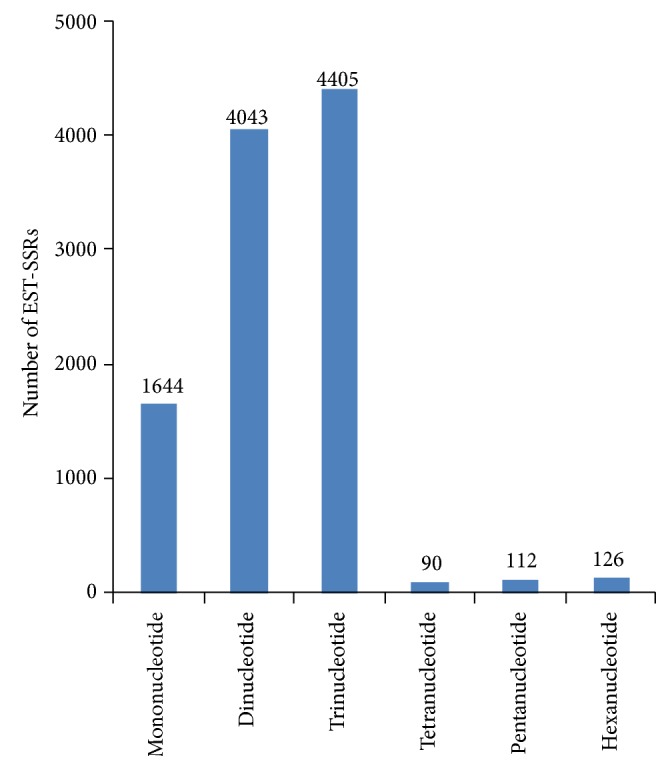
EST-SSR statistics.

The size of SSR repeat units ranged from one to six. The number of SSRs with each repeat unit was found to be quite different. The SSRs with tri- and dinucleotide repeat motifs were the most common (4,405, 42.27%; 4,043, 38.80%, resp.), followed by mono- (1,644, 15.78%), hexa- (126, 1.21%), penta- (112, 1.07%) and tetra- (90, 0.86%) nucleotide repeat motifs (Figure 2). The most common two repeat motif types accounted for 81.07% of the total SSRs detected, and the rest repeat motifs types only accounted for 18.93%.

The iterate number of repeat units in an EST-SSR ranged from 4 to 25. The occurrence frequency of EST-SSTs with different iterate numbers was found to be unequal either. EST-SSRs with iterate number of 5 (2832, 27.18%) were the most common ones, followed by 6 (2739, 26.29%), 7 (1368, 13.13%), 8 (703, 6.75%), 12 (542, 5.20%), and 9 (480, 4.61%) (Table s3). A dinucleotide containing EST-SSRs with a maximum of 25 repeat units was identified. For EST-SSRs with more than 10 repeat units, the mononucleotide repeat motifs were the most abundant, accounting for 93.46% of these EST-SSRs. The lengths of EST-SSR sequences ranged from 12 to 65 bp (Table s4). The longest one is a pentanucleotide containing EST-SSR with 65 bp in length. The lengths of most EST-SSRs are from 12 to 20 bp, accounting for 91.47% of the total EST-SSRs, followed by EST-SSRs with 21–30 bp in length (874 SSRs, 8.39%). Only 13 EST-SSRs were identified with over 30 bp, accounting for 0.12% of the total EST-SSRs.

**Figure 3:**
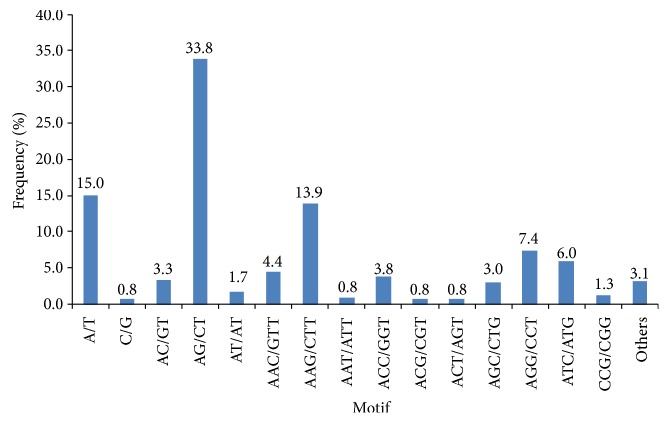
Frequency distribution of EST-SSRs according to motif sequence types.

A total of 124 EST-SSR motifs were identified, including 2 mono-, 3 di-, 10 tri-, 13 tetra-, 33 penta-, and 63 hexanucleotide repeat units containing EST-SSRs. The dominant motif identified in our EST-SSRs was AG/CT (3,519, 33.8%), followed by A/T (1,562, 15.0%), AAG/CTT (1,445, 13.9%), AGG/CCT (776, 7.4%), ATC/ATG (627, 6.0%), AAC/GTT (392, 4.4%), ACC/GGT (392, 3.8%), AC/GT (349, 3.3%), and AGC/CTG (317, 3.0%) (Figure 3). The other 115 motifs have low frequency, accounting only for 9.3% of total EST-SSRs.

**Figure 4:**
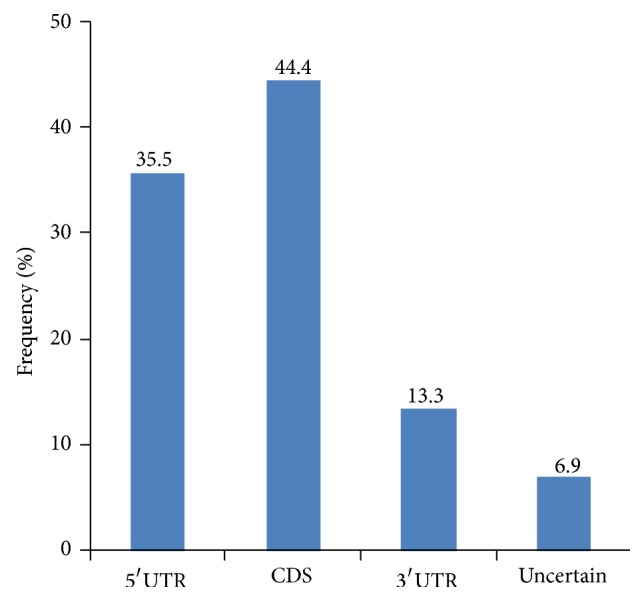
Frequency distribution of EST-SSRs based on locations.

Physical locations of the EST-SSRs were assigned by searching against the nonredundant (nr) protein database of NCBI (http://www.ncbi.nlm.nih.gov/) and the* Brassica* database (http://brassicadb.org/brad/) using BLASTX. Our results showed that 4329 EST-SSRs (44.4%) were located in coding regions (CDSs), 3456 (35.5%) in 5′-UTRs, and 1297 (13.3%) in 3′-UTRs (Figure 4, Table s4). Locations of the remaining 672 EST-SSRs (6.9%) were not successfully assigned (Figure 4, Table s4). For the EST-SSRs localized in the CDS region, trinucleotide repeats were the most common ones, accounting for 62.72% of the total EST-SSRs localized in this region, followed by dinucleotide repeats (897, 20.72%), mononucleotide repeats (325, 7.51), and compound formation (287, 6.63%) (Table s4). Dinucleotide repeats (1909, 55.24%) were the dominant types in 5′-UTRs, followed by trinucleotide repeats (730, 21.12%), mononucleotide repeats (483, 13.98%), and compound formation ones (214, 6.19%) (Table s4). Mono-, di-, and trinucleotide repeat EST-SSRs were the top three types found in 3′-UTRs, accounting for 35.08%, 30.07%, and 28.60% of the total EST-SSRs localized in these regions, respectively.

### 3.3. New EST-SSRs Identification

The EST-SSRs and the flanking sequences (50 bp on each side) were aligned to the* Brassica rapa* (Chiifu-401) reference genome (http://brassicadb.org/brad/) using BLASTN to determine their physical positions. New EST-SSRs were identified by comparing with the earlier reported SSRs in the SSR marker database for* Brassica* (http://oilcrops.info/SSRdb). A total of 2744 new EST-SSRs (26.3%) were identified in the study. Of the 7676 known SSRs (73.6%), 2317 EST-SSRs (22.2%) show polymorphism with different repeat numbers, and 5359 (51.4%) were exactly the same with the earlier reported SSRs based on the* Brassica rapa* (Chiifu-401) genomic sequence [25] (Table s2).

### 3.4. Primer Design and Evaluation of EST-SSRs in Chinese Cabbage

A total of 7877 PCR primer pairs from the unique sequences flanking 1561 EST-SSR loci were designed according to the criteria described in Section 2 using primer 3 (Table s5). For each EST-SSR locus, a maximum of 5 alternative primer pairs was designed. The other 8859 EST-SSRs, which had no appropriate PCR primer pairs designed as their flanking sequences, did not fulfill the primer design criteria mentioned above. For the 1561 EST-SSRs with PCR primers designed, PCR primers of those aforementioned 24 loci with *n* ≥ 20 bp were selected for primer synthesis and amplification evaluation in Chinese cabbage FuShanBaoTou. Nineteen (79.2%) of these 24 EST-SSR loci successfully yielded PCR amplicons in FuShanBaoTou. We sequenced these nineteen PCR amplicons and found that the amplicons in thirteen loci were exactly the same as expected; two were longer than the expected size, and four were shorter (Table 3). Size deviation of five EST-SSRs loci with the expected sizes (BR-es6, BR-es7, BR-es8, BR-es12, and BR-es18) was due to the variations of SSR repeat motifs (Table s6). One amplicon (BR-es16) deviated from the expected sizes and had an additional 86 bp containing a (TC)_9_ motif near the SSR repeat motif region (Table s6).

### 3.5. Validation of Polymorphism of EST-SSRs

**Figure 5:**
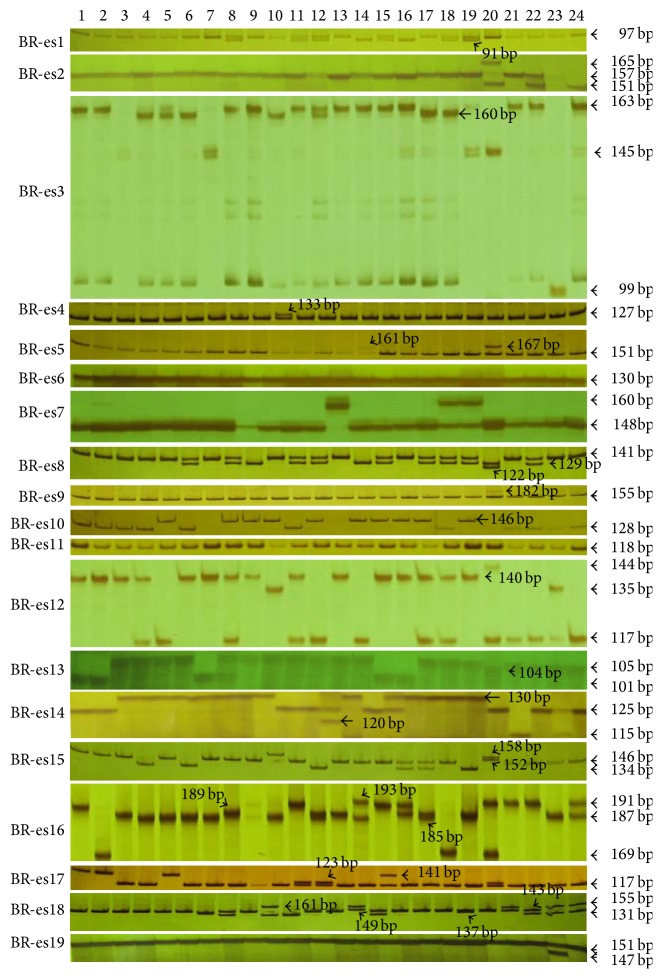
PCR products amplified by nineteen effective EST-SSR primer pairs in twenty-four cultivars of Chinese cabbage. The order of DNA samples from lane 1 to lane 24 within each primer pair image panel is 682, GuangDongZao, ZaoHuangBai, Z61-8, FuShanBaoTou, Li-3, 212-7, TianJinQingMaYe, KuaiCai number 6-5, JinHuangXiaoBaiCai, SiJiXiaoBaiCai, SiJiHuangYangXiaoBaiCai, PinZao number 1, HanYuTeXuanHuangXin, QuanNengSiJiKuaiCai, JingYouXiaoBaiCaiKuaiCai, GaoLiWaWaCai, KeYiXiaWaWa, JinNuoChunQiuWaWaCai, SiJiLvGanXiaoKuCai, YouLv157, ShuYaoYouCai, DeGaoYouLiangQingGengCai, and QingXiuF1QingGengCai. PCR products amplified by BR-es2, BR-es3, BR-es6, BR-es7, BR-es12, BR-es13, BR-es14, BR-es16, and BR-es19 primer pairs were separated on 6% denaturing polyacrylamide gels, while those amplified by BR-es1, BR-es4, BR-es5, BR-es8, BR-es9, BR-es10, BR-es11, BR-es15, BR-es17, and BR-es18 primer pairs were separated on 12% nondenaturing polyacrylamide gels.

Nineteen effective primer pairs were used for polymorphism validation for these aforementioned 24 Chinese cabbage cultivars. The results showed that 17 loci (89.5%) were polymorphic (Figure 5). A total of 56 alleles at the 17 polymorphic loci were identified and the average number of alleles per SSR locus was 3.29 with a range between 2 and 6. A maximum of 6 alleles was detected for BR-es16 and BR-es18 loci. BR-es6 and BR-es11 had no polymorphic allele in all 24 cultivars in this study (Figure 5, Tables 3 and s4). Of the 17 polymorphic loci, twelve loci were polymorphic in all cultivars of* B. pekinensis *L. and* B. chinensis *L. Three loci (BR-es2, BR-es9, and BR-es19) had no polymorphism in the cultivars of* B. pekinensis *L. but had polymorphism in the cultivars of* B. chinensis* L., while two loci (BR-es4 and BR-es7) were polymorphic in the cultivars of* B. pekinensis *L. but were not polymorphic in the cultivars of* B. chinensis *L. (Figure 5, Table s8).

We sequenced the polymorphic alleles of the 17 polymorphic loci and found that polymorphisms of 9 loci (BR-es1, BR-es4, BR-es7, BR-es8, BR-es10, BR-es14, BR-es17, BR-es18, and BR-es19) were because of different iterate numbers of SSR repeat motifs. In another 6 polymorphic loci (BR-es2, BR-es3, BR-es12, BR-es13, BR-es15, and BR-es16), the most polymorphic alleles were found in the repeat motifs with additional changes in other regions (Table s7). For example, compared with the allele BR-es3-160 bp in FuShanBaoTou, the polymorphic alleles BR-es3-163 bp and 145 bp had different iterate numbers of the TAG/ATC repeat motif, while the polymorphic allele 99 bp had not only a different number of the repeat motif, but also a deletion in another region (Table s7). The other two polymorphic loci, BR-es5 and BR-es9, had polymorphisms that are not related with the repeat numbers of SSR motifs (Table s7).

## 4. Discussion

### 4.1. High-Throughput RNA Sequencing Provides Substantial Knowledge for EST-SSRs

Illumina paired-end RNA sequencing is one of the fast immerging next-generation sequencing (NGS) technologies. Because of its advantages in high-throughput, high accuracy, and low cost, Illumina paired-end sequencing has been widely used for* de novo* transcriptome sequencing and assembly and transcriptome quality and quantity analysis in many plants [37, 38, 42, 43]. In our previous study, the transcriptome of rosette and folding leaves in Chinese cabbage was analyzed using the Illumina paired-end RNA sequencing technology, and abundant clean reads and ESTs with high quality were obtained [37]. The large quantity of clean reads would increase coverage depth of transcriptome nucleotide, enhance sequencing accuracy, and provide useful information for developing new tools for genetic mapping and molecular breeding of Chinese cabbage. In this study, we further assembled the clean reads into contigs and unigenes from the RL and FL libraries, respectively. The parameters for both contigs and unigenes between the two libraries had no significant differences (Table 1), indicating our Illumina sequencing solutions have high reliability and reproducibility. The unigenes of the two libraries were further assembled and a total of 51,694 nonredundant unigenes were obtained from the 40.7 Mb sequence data. We discovered more nonredundant unigenes than those in previous studies [35, 36], which represent a large portion of the Chinese cabbage transcriptome and are important for a comprehensive understanding of EST-SSRs.

### 4.2. Frequency and Distribution of EST-SSRs in Chinese Cabbage

A total of 10,420 SSRs with over 12 bp were identified from the deep transcriptome sequence dataset of Chinese cabbage. About 16.6% of the unigenes have SSRs. The frequency of occurrence of SSRs is slightly higher than those reported in previous studies on Chinese cabbage (about 8.4–15.6%) [20, 34–36] and also higher than those of other dicotyledonous species such as peanut (6.8%) [44], sweetpotato (8.2%) [21], sesame (8.9%) [43], pigeonpea (7.6%) [45], grapes (2.5%) [46], pepper (4.9%) [47], and flax (3.5%) [48], but it is lower than those of coffee (18.5%) [49], radish (23.8%) [38], and caster bean (28.4%) [50]. Detection of EST-SSRs depends on a number of factors such as genome structure [51], tools and parameters for EST-SSRs detection and exploration [43], and size of dataset for unigene assembly [27].

The frequency of SSRs with different sizes of repeat units is not evenly distributed in plants. Previous studies showed dinucleotide SSR loci are the most abundant class in safflower [52], pigeonpea [45], and sesame [43], whereas trinucleotide repeats are the most frequent ones in barley [53], sweetpotato [21],* Jatropha curcas* [54], iris [55], pepper [47], caster bean [50], flax [48],* Cucurbita pepo* [56], and radish [38]. In ramie [57] and wheat [58], dinucleotide and trinucleotide repeat motifs are the most two abundant types. In the present study, trinucleotide (4405, 42.3%) was found to be the most common repeat motif class in Chinese cabbage, followed by dinucleotide (4043, 38.8%) (Figure 2). It is consistent with previous reports for SSRs identification from unigenes of Chinese cabbage [20]. However, on the genomic level, of Chinese cabbage, dinucleotide is the most common repeat motif, followed by trinucleotide [25].

We found the most dominant mononucleotide repeat motif in Chinese cabbage was A/T (1,562, accounting for 15.0% of the total EST-SSRs), which is consistent with previous reports for Chinese cabbage [25] and for other plants such as* Arabidopsis* [59], rice [59], wheat [60], radish [38], castor bean [50],* Gossypium raimondii *[61], oil palm [62], and eggplant [63]. For dinucleotide motif, AG/CT was the most common repeat motif, accounting for 87.0% of the total dinucleotide EST-SSRs. It is in close agreement with the results in previous studies for genic SSRs in Chinese cabbage [20, 36] and those in most other plants such as sweetpotato [21], iris [55], sesame [43], and radish [38]. The AG/CT repeat motif was also the most dominant repeat among all the EST-SSRs identified in this study, accounting for 33.8% of the total EST-SSRs. However, for genomic-SSRs in Chinese cabbage, AT/TA is the most common dinucleotide motif [25]. The AAG/CTT (1,445, 13.9%) motif was the most frequent motif among trinucleotide EST-SSRs in the study, which is consistent with the results in previous studies in Chinese cabbage [25, 36] and many dicot species, for example,* Arabidopsis* [64], soybean [65], peanut [44], sweetpotato [21], radish [38], and sesame [43]. In many monocot species such as maize, barley, and sorghum [66, 67], CCG/GGC is the most dominant trinucleotide repeat motif. It is considered a specific feature of monocot genomes due to the high GC content in monocot genomes [68].

### 4.3. New EST-SSRs Identification

Of all 10420 EST-SSRs identified in this study, more than 70% have been identified and presented in the SSR marker database (http://oilcrops.info/SSRdb), among which over half were exactly the same with the earlier reported SSRs based on the* Brassica rapa* (Chiifu-401) genomic sequence (Table s2) [25]. It demonstrates that our method is highly reliable for EST-SSR identification. 2317 EST-SSRs (22.2%) with polymorphism in different repeat numbers could further be used for identification of Chiifu-401 and FuShanBaoTou and for genetic linkage map constructions using these two cultivars as parents. A total of 2744 new EST-SSRs (26.3%) were identified in the study, which, in combination with previously discovered EST-SSRs, could be used for high-density genetic linkage map construction, gene/QTL mapping, cultivar identification, and so forth.

### 4.4. High Polymorphism of Chinese Cabbage EST-SSRs

In the present study, 79.2% of the EST-SSRs primer pairs selected for primer evaluation successfully generated high quality amplicons, indicating that the ESTs from the high-throughput RNA sequencing of Chinese cabbage transcriptome are suitable for specific primer design. The unsuccessfully designed primer pairs may be due to splice sites, large introns, chimeric primer(s), or poor quality sequences [27]. We sequenced all PCR amplicons in Chinese cabbage FuShanBaoTou yielding 19 successful primer pairs. We found that all amplicons contained the expected SSRs and the SSRs in 13 amplicons were exactly the same as predicted (Table s6). The deviation of EST-SSR PCR amplicons from the expected size is likely due to the presence of introns, large insertions or repeat number variations, a lack of specificity, or assembly errors [43]. In the present study, we found five of six amplicons with unexpected sizes had different iterate number of SSR repeat units, while the other one had a 86 bp insertion near the expected SSR repeat motif region (Table s6). These results suggested that the unigenes assembled from the high-throughput RNA sequencing of Chinese cabbage transcriptome are reliable, and the EST-SSRs identified in our dataset could be used for further studies, such as genetic mapping and cultivar identification.

Most of the EST-SSR loci (accounting for 89.5% of the tested loci) were found to be polymorphic among the 24 tested cabbage cultivars. The mean number of alleles per SSR locus was 3.29 with a range between 2 and 6 (Table 3), indicating that polymorphism of EST-SSRs in Chinese cabbage is relatively high. Most of the polymorphisms of the tested EST-SSR loci are due to the variations of SSR repeat motifs in this study. There were only two loci where the polymorphisms were not related to the SSR repeat motif variations (Table s6). The results indicate that the EST-SSRs identified and the PCR primers designed in this study could further be used for constructing high-density genetic linkage maps, mapping quantitative trait loci, assessing germplasm polymorphism and evolution, marker-assisted selection, and cloning functional gene in Chinese cabbage.

In summary, we assembled a large set of clean reads with high quality derived from the Chinese cabbage transcriptome using high-throughput RNA sequencing technology with a Solexa/Illumina platform. A total of 51,694 nonredundant unigenes were obtained from 40.7 Mb sequence data, providing substantial knowledge for EST-SSR identification and characterization. 10,420 EST-SSRs were identified and characterized, and PCR primer pairs for 1561 EST-SSRs were designed. By comparing with previously reported SSRs in the SSR marker database for* Brassica* (http://oilcrops.info/SSRdb), we identified a total of 2744 new EST-SSRs. Primer pairs for 24 EST-SSRs were selected for primer evaluation, and 79.2% of the 24 EST-SSR loci successfully generated high quality amplicons. Among the effective primers, 89.5% of them showed polymorphism in 24 cultivars of Chinese cabbage. The EST-SSRs developed in this study, in combination with previously reported EST-SSRs, will provide valuable resources for constructing high-density genetic linkage maps, mapping quantitative trait loci, assessing germplasm polymorphism and evolution, marker-assisted selection, and cloning functional gene in Chinese cabbage. To our knowledge, this is the first successful attempt to develop large quantity of EST-SSRs with high quality based on the transcriptome of Chinese cabbage using high-throughput RNA sequencing technology.

## Supplementary Material

Table S1: Sequences of all unigenes.
Table S2: Detail descriptions of all the EST-SSRs identified in the study.
Table S3: Distribution of EST-SSRs based on the number of repeat units.
Table S4: Distribution of EST-SSRs on different regions based on the number of repeat units.
Table S5: Primers of EST-SSR identified in the study.
Table S6: Expected and validated sequences of the five deviation EST-SSR markers.
Table S7: PCR primer pairs, polyporphism alleles and their sequences of the 19.
Table S8: Genetypes of the the 19 EST-SSRs in the 24 Chinese cabbage cultivars used for detecting the polymorphism.

## Figures and Tables

**Table 1:** Overview of the sequencing and assembly.

	Sample	Total number	Total length (nt)	Average length (nt)	N50	Total consensus sequences	Distinct clusters	Distinct singletons
Contig	RL	99,684	33,205,708	333	531	—	—	—
	FL	95,441	32,596,297	342	536	—	—	—

Unigene	RL	46,294	32,729,586	707	1000	46,294	19,512	26,782
	FL	48,473	32,971,187	680	980	48,473	19,749	28,724
	All	51,694	40,724,256	788	1154	51,694	23,850	27,844

**Table 2:** Summary of EST-SSR searching results.

Searching items	Numbers
Total number of sequences examined	51694
Total size of examined sequences (bp)	40724256
Total number of identified SSRs	10420
Number of SSR-containing sequences	8571
Number of sequences containing more than one SSR	1502
Number of SSRs present in compound formation	666
% EST-SSRs	16.6%

**Table 3:** Details of 19 EST-SSRs that successfully yielded PCR amplicons in FuShanBaoTou.

Code	EST-SSR name	Motif	Product size expected (bp)	Product size validated (bp)	SSR location	Number of alleles
BR-es1	CL3455.Contig1_All-2	(TC)11	97	97	CDS	2
BR-es2	CL4114.Contig2_All-2	(TCA)7	157	157	3-UTR	3
BR-es3	CL2525.Contig4_All-1	(TAG)9	160	160	CDS	4
BR-es4	Unigene10387_All-1	(CTC)9	127	127	CDS	2
BR-es5	CL7077.Contig2_All-1	(AATC)5	153	153	3-UTR	3
**BR-es6**	Unigene16359_All-1	(AACC)5	**134**	**130**	5-UTR	1
**BR-es7**	CL4685.Contig1_All-1	(CCTT)6	**160**	**148**	3-UTR	2
**BR-es8**	CL5247.Contig3_All-1	(TTTC)6	**133**	**141**	CDS	3
BR-es9	CL3462.Contig4_All-1	(AATCG)4	155	155	CDS	2
BR-es10	CL5726.Contig2_All-2	(TCTCT)4	146	146	5-UTR	3
BR-es11	Unigene6713_All-1	(AAAAC)4	118	118	CDS	1
**BR-es12**	CL7282.Contig2_All-1	(GAGGA)5	**140**	**117**	CDS	4
BR-es13	Unigene2970_All-1	(GAACT)5	106	106	3-UTR	3
BR-es14	Unigene8739_All-1	(GATTT)5	130	130	5-UTR	4
BR-es15	CL5873.Contig4_All-2	(CCCTAA)4	146	146	3-UTR	4
**BR-es16**	Unigene14449_All-1	(CTCAAG)5	**99**	**185**	CDS	6
BR-es17	Unigene5096_All-1	(ACTCCC)5	141	141	CDS	3
**BR-es18**	CL4691.Contig2_All-1	(GATGGT)7	**155**	**117**	CDS	6
BR-es19	Unigene13507_All-1	(ATTTG)4	152	152	CDS	2

EST-SSRs shown in bold have sizes different from the expected sizes.
